# Characteristics and management of headache among psychiatric outpatients at a Japanese general hospital: A retrospective study with an exploratory CGRP case series

**DOI:** 10.1002/pcn5.70235

**Published:** 2025-10-30

**Authors:** Kyohei Otani, Nobuyasu Imbe, Ryota Shindo

**Affiliations:** ^1^ Department of Psychiatry Kakogawa Central City Hospital Kakogawa Hyogo Japan

**Keywords:** CGRP‐related agents, depression, migraine, psychiatric disorders, tension‐type headache

## Abstract

**Aim:**

Headache is one of the most frequent somatic complaints in psychiatric practice and is often attributed to underlying mental disorders. However, primary headache disorders—particularly migraine and tension‐type headache (TTH)—commonly coexist with psychiatric conditions. Evidence from psychiatric outpatient settings remains limited.

**Methods:**

We conducted a retrospective chart review of all psychiatric outpatients who visited our 600‐bed regional general hospital between April 1, 2023, and March 31, 2024. Among 2525 patients, we identified 360 individuals with headache‐related insurance diagnoses and extracted data on headache labels, treating departments, and prescribed medications. For calcitonin gene–related peptide (CGRP)‐targeted monoclonal antibodies, we extended the observation period to March 31, 2025, to describe an exploratory case series including additional prescriptions.

**Results:**

Of 2525 psychiatric outpatients, 360 (14.3%) carried a headache‐related insurance diagnosis. The most frequent labels were “headache” (203/360, 56.4%), migraine (92/360, 25.6%), and TTH (46/360, 12.8%); cluster headache and medication‐overuse headache (MOH) were each recorded in 1/360 (0.3%). Headache care was most often delivered within psychiatry (153/360, 42.5%), followed by neurology (42/360, 11.7%), neurosurgery (40/360, 11.1%), general internal medicine (28/360, 7.8%), and rheumatology/collagen‐vascular disease (15/360, 4.2%). Commonly documented agents included nonsteroidal anti‐inflammatory drug (NSAIDs) (40/360, 11.1%), acetaminophen (38/360, 10.6%), triptans (23/360, 6.4%), Japanese Kampo formulas (16/360, 4.4%), and CGRP monoclonal antibodies (6/360, 1.7%). At the agent level, acetaminophen (*n* = 38), loxoprofen (*n* = 33), zolmitriptan (*n* = 14), goreisan (*n* = 8), sumatriptan (*n* = 6), kakkonto (*n* = 6), diclofenac (*n* = 4), valproic acid (*n* = 4), and naratriptan (*n* = 3) were among the most frequently listed. In the exploratory CGRP analysis (total seven patients through March 31, 2025), six were women; the mean age was 48.4 ± 9.2 years. Psychiatric comorbidities were heterogeneous, including eating disorder, bipolar disorder, post‐traumatic stress disorder, dysthymia with social anxiety disorder, schizophrenia, autism spectrum disorder, and neurotic depression. All cases experienced headache improvement; two required switching to another CGRP agent for recurrent attacks yet maintained benefit. One patient temporarily discontinued due to a rash before resuming a different CGRP agent. In contrast, medium‐term changes in mood/anxiety were limited.

**Conclusion:**

In a psychiatric outpatient cohort, primary headaches were common and frequently managed within psychiatry. CGRP‐targeted therapy yielded headache relief even under psychiatric comorbidity, while psychiatric symptoms did not uniformly improve, underscoring the need for parallel mental‐health interventions alongside headache‐specific care. Strengthening cross‐specialty pathways and early headache evaluation within psychiatry are warranted.

## Graphical abstract

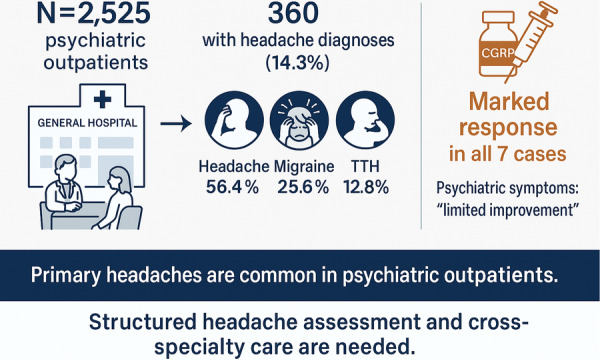

## INTRODUCTION

Headache is among the most common physical complaints encountered in psychiatric practice. Prior research demonstrates that primary headache disorders, especially migraine and tension‐type headache (TTH), have high comorbidity with depression and anxiety in both adults and youth.^1^, ^2^, ^3^, ^4^ The relationship between psychiatric disorders and headache is thought to be bidirectional. Nevertheless, much of the literature originates from neurology or primary care settings. Data specifically describing the profiles and management of headache among psychiatric outpatients at general hospitals are scarce.

In routine psychiatric care, headaches may be overlooked or undertreated when overshadowed by prominent psychiatric symptoms, potentially delaying adequate headache assessment and guideline‐based management. This phenomenon may lead to suboptimal outcomes and increased healthcare utilization.

This study aimed to describe (i) the clinical and management characteristics of psychiatric outpatients carrying headache‐related insurance diagnoses at a large regional general hospital and (ii) an exploratory case series of patients receiving calcitonin gene–related peptide (CGRP) monoclonal antibodies within psychiatric care. We hypothesized that primary headaches are common in psychiatric clinics and that, even under psychiatric comorbidity, CGRP therapy would improve headache metrics while psychiatric symptoms may show modest or inconsistent change.

## METHODS

### Setting and participants

Kakogawa Central City Hospital is a 600‐bed regional general hospital with 33 departments, including neurology, neurosurgery, obstetrics/gynecology, pediatrics, otorhinolaryngology, general/internal medicine, and psychiatry. The psychiatry department accepts patients across the perinatal to geriatric spectrum.

We retrospectively screened all psychiatric outpatients between April 1, 2023, and March 31, 2024 (index period). Among 2525 unique patients, we included those with headache‐related insurance diagnoses documented in the electronic medical record (EMR), yielding 360 cases for analysis.

### Data extraction

We recorded the following: (1) headache‐related insurance diagnoses, (2) psychiatric diagnoses, (3) treating department for headache, and (4) prescribed headache medications. When multiple headache labels or multiple drugs were present, we simplified analysis by selecting the label considered most clinically relevant (based on symptom severity and the presumed primary diagnosis) and by summarizing representative drug classes and frequently used agents.

For the CGRP subgroup, we extended the capture period to April 1, 2023, to March 31, 2025, to describe all psychiatric outpatients who received CGRP monoclonal antibodies, and we summarized sex/age, psychiatric comorbidities, treatment course (switching/temporary discontinuation), and qualitative changes in psychiatric symptoms.

Headache diagnoses were assigned according to the International Classification of Headache Disorders, 3rd edition (ICHD‐3). Psychiatric diagnoses were made in accordance with the DSM‐5 (American Psychiatric Association), based on routine clinical assessments documented by board‐certified psychiatrists.

### Operational definitions

We used “medication‐overuse headache (MOH)” per ICHD‐3 in place of the older term “drug overuse headache.” The term “Kampo (traditional Japanese) formulas” denotes formulations such as goreisan and kakkonto. “Ditan” refers to lasmiditan. Because this was a service evaluation/retrospective audit, standardized headache severity scales (e.g., headache impact test‐6 [HIT‐6], migraine disability assessment scale [MIDAS]) or standardized psychiatric symptom scales (e.g., PHQ‐9, generalized anxiety disorder‐7 [GAD‐7]) were not uniformly administered and were therefore not analyzed quantitatively.

### Ethics

The study protocol was approved by the Institutional Review Board of Kakogawa Central City Hospital (approval No. 2024‐53). As a retrospective study using de‐identified data, the requirement for individual informed consent was waived.

### Statistical analysis

We performed descriptive analyses only. Categorical variables are presented as counts (percentages). Continuous variables, where applicable, are summarized as mean ± SD. No hypothesis tests were planned a priori given the exploratory nature and heterogeneous documentation.

## RESULTS

**Figure 1:**
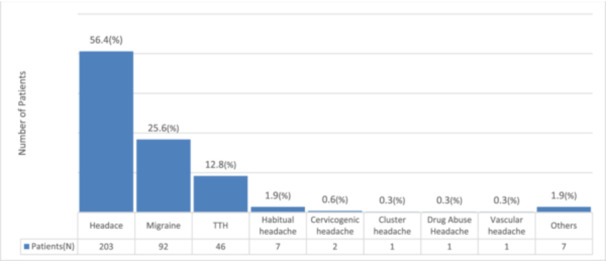
Distribution of headache‐related insurance diagnoses among psychiatric outpatients (*N* = 360). *Note*: Diagnoses are based on insurance billing records in the electronic medical record system and may not correspond precisely to ICHD‐3 classifications. The high proportion of unspecified “headache” diagnoses reflects common documentation practices in routine psychiatric outpatient care. TTH, tension‐type headache.

Among 2525 psychiatric outpatients during the index period, 360 (14.3%) had a headache‐related insurance diagnosis. The cohort comprised 266 females (73.9%) and 94 males (26.1%), with a mean age of 55.0 ± 18.7 years (range 15–96 years). The specific headache‐related insurance diagnoses and their frequencies were as follows: “Headache” (*n* = 203, 56.4%), “Migraine” (*n* = 92, 25.6%), “Tension‐type headache” (*n* = 46, 12.8%), “Habitual headache” (*n* = 7, 1.9%), “Cervicogenic headache” (*n* = 2, 0.6%), “Cluster headache” (*n* = 1, 0.3%), “Medication‐overuse headache” (*n* = 1, 0.3%), “Vascular headache” (*n* = 1, 0.3%), and “Other headache‐related diagnoses” (*n* = 7, 1.9%). These diagnostic labels were extracted from the EMR for insurance billing purposes, which do not always correspond precisely to ICHD‐3 classifications in routine psychiatric outpatient care (Figure 1).

**Figure 2:**
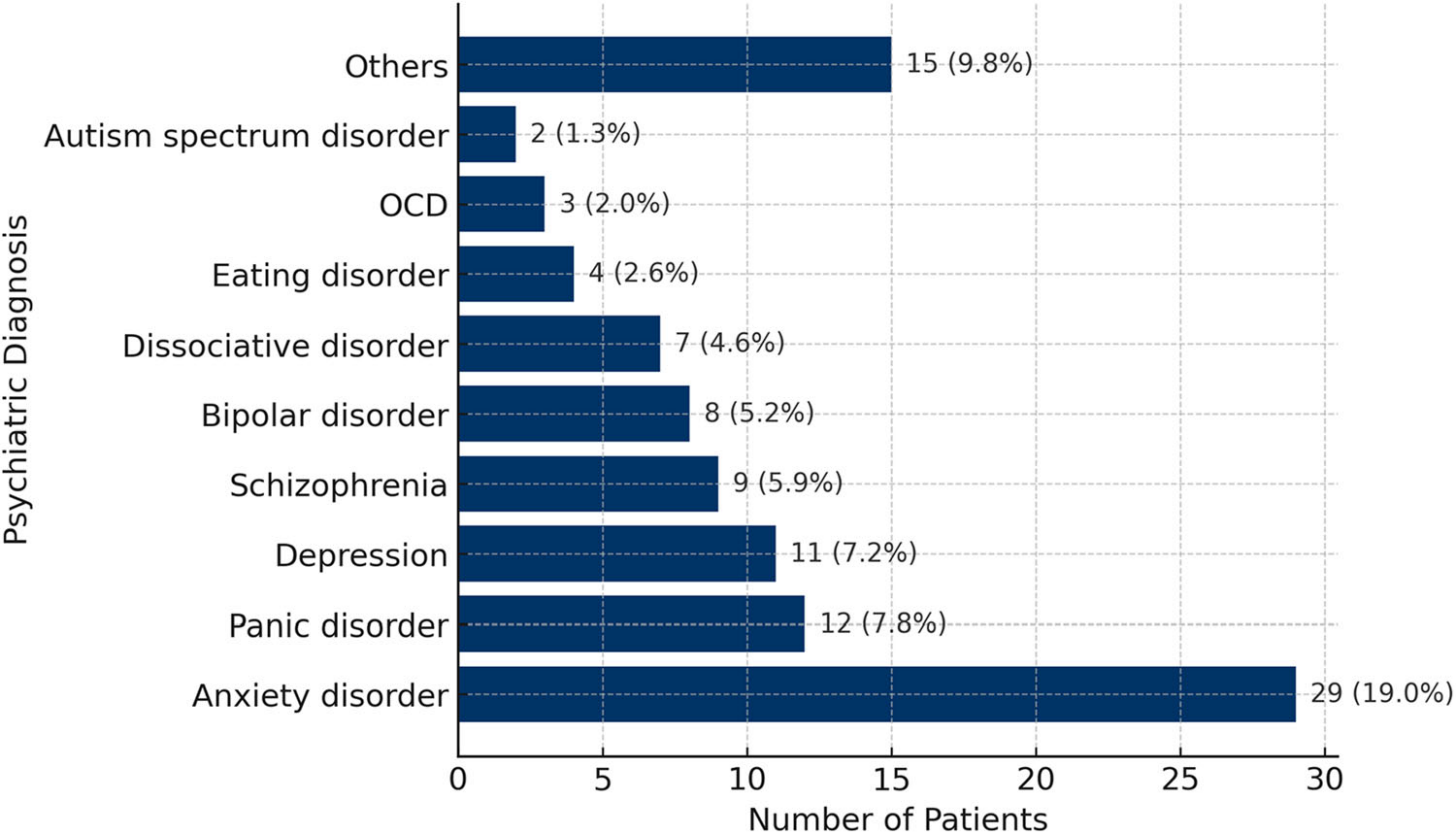
Distribution of psychiatric diagnoses among patients receiving headache care in psychiatry (*N* = 153). *Note*: Patients may have multiple concurrent psychiatric diagnoses. Therefore, percentages sum to more than 100%. OCD, obsessive‐compulsive disorder.

Psychiatric diagnoses were diverse and included anxiety and panic disorders as the most frequent categories, alongside depressive disorders, dissociative disorders, intellectual disability, autism spectrum disorder, adjustment disorder, dementia, schizophrenia, eating disorders, obsessive–compulsive disorder, bipolar disorder, epilepsy, and others. Among the 153 patients who received headache care within the psychiatry department, the most common psychiatric diagnoses were anxiety disorders (*n* = 29, 19.0%), panic disorder (*n* = 12, 7.8%), and depression (*n* = 11, 7.2%) (Figure 2).

**Figure 3:**
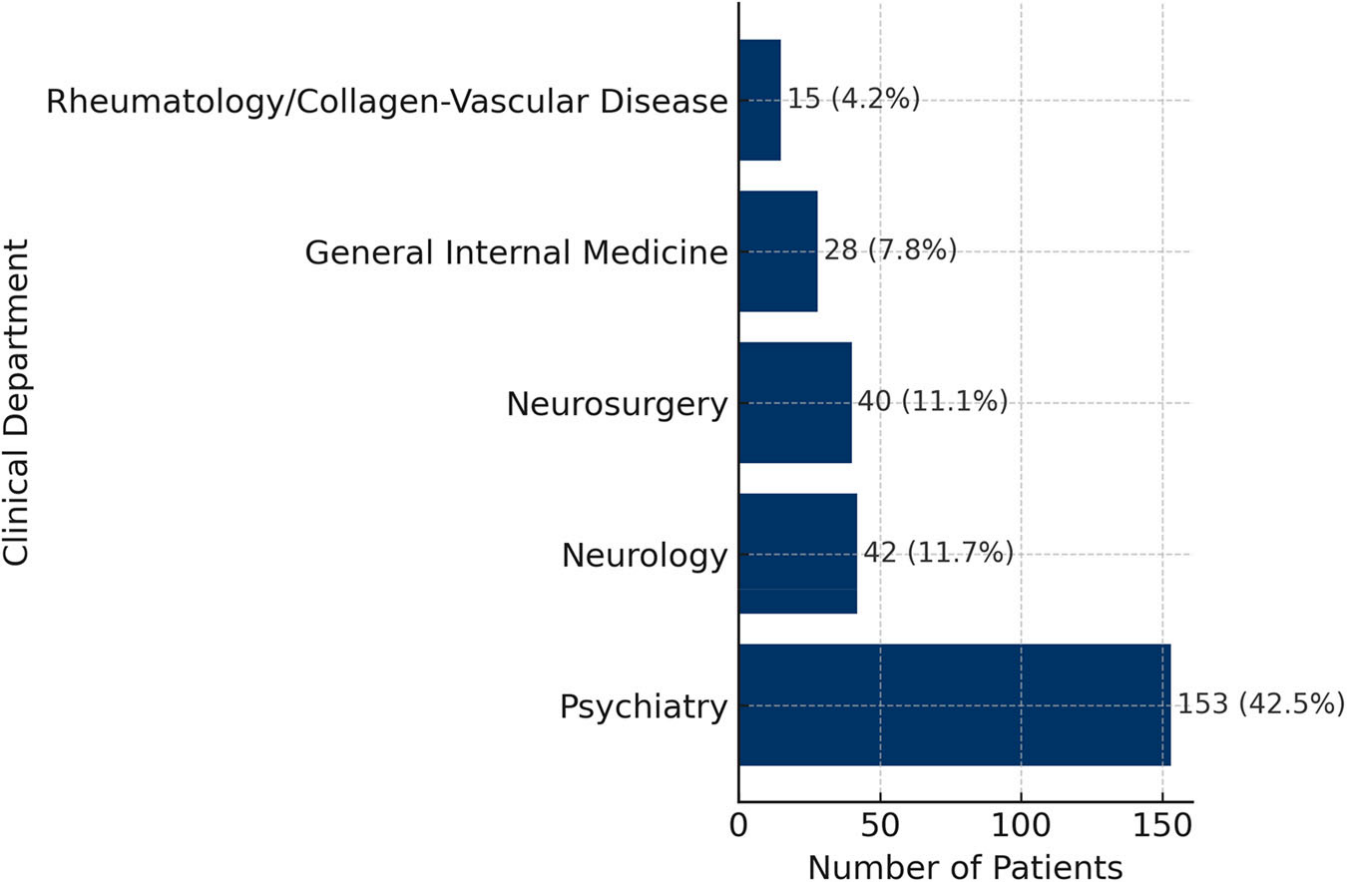
Clinical departments providing headache care (*N* = 360). Clinical departments initially consulted for headache complaints (e.g., psychiatry, neurology, and internal medicine), illustrating referral patterns within the general hospital.

Headache care was provided most often within psychiatry: 153/360 (42.5%), followed by neurology 42/360 (11.7%), neurosurgery 40/360 (11.1%), general internal medicine 28/360 (7.8%), and rheumatology/collagen‐vascular disease 15/360 (4.2%) (Figure 3).

Documented medications included NSAIDs (40/360, 11.1%), acetaminophen (38/360, 10.6%), triptans (23/360, 6.4%), Kampo formulas (16/360, 4.4%), CGRP monoclonal antibodies (6/360, 1.7%), antiepileptic agents (4/360, 1.1%), muscle relaxants (3/360, 0.8%), antidepressants prescribed for headache (2/360, 0.6%), lasmiditan (1/360, 0.3%), and ergotamine (1/360, 0.3%); no specific headache medication was recorded in 26/360 (7.2%).

At the agent level, acetaminophen (*n* = 38), loxoprofen (*n* = 33), zolmitriptan (*n* = 14), goreisan (*n* = 8), sumatriptan (*n* = 6), kakkonto (*n* = 6), diclofenac (*n* = 4), valproic acid (*n* = 4), and naratriptan (*n* = 3) were among the most frequently listed.

Within the index period, six patients received CGRP monoclonal antibodies. Extending the observation through March 31, 2025, identified one additional case (total *n* = 7). Six were women, 85.7%, and the mean age was 48.4 ± 9.2 years (range 33–64 years).

Psychiatric comorbidities were heterogeneous—eating disorder, bipolar disorder, post‐traumatic stress disorder, dysthymia with social anxiety disorder, schizophrenia, autism spectrum disorder, and neurotic depression—and no single psychiatric diagnosis predominated.

Among the seven patients who received CGRP monoclonal antibodies (Table 1), all achieved marked headache response defined as substantial patient‐reported reduction in headache frequency and/or severity. Four patients continued CGRP therapy at the last follow‐up, while one patient was successfully transitioned to conventional prophylactic medication after achieving sustained. Treatment courses varied: 4 patients (57%) maintained sustained benefit throughout follow‐up, 2 patients (29%) experienced recurrence of attacks requiring switch to an alternative CGRP agent with subsequent resumption of benefit, and 1 patient (14%) temporarily discontinued treatment due to rash before resuming with a different CGRP agent. In contrast to consistent headache improvement, psychiatric symptoms showed limited change: 6 of 7 patients (86%) reported unchanged or worsened psychiatric symptoms during the observation period. No serious adverse events were recorded.

**Table 1:** Clinical characteristics and outcomes of psychiatric outpatients treated with calcitonin gene–related peptide (CGRP) monoclonal antibodies (*N* = 7).

Case	Age	Sex	Psychiatric diagnosis	Primary psychotropic	CGRP agent(s)	Headache response	Psychiatric trajectory	Time to clinical response (weeks)	CGRP treatment duration (months)	Follow‐up duration (months)
1	50	F	Social anxiety disorder	Venlafaxine	Fremanezumab → galcanezumab^a^	Marked response	Unchanged	**4**	**14**	**20**
2	33	F	Schizophrenia	Blonanserin patch	Galcanezumab → fremanezumab^b^	Marked response → relapse^b^	Unchanged	**4**	**15**	**15** b
3	49	M	Bipolar disorder	Brexpiprazole	Galcanezumab → erenumab^b^	Marked response	Unchanged	**4**	**15**	**15** b
4	45	F	Eating disorder	Quetiapine	Galcanezumab	Marked response	Worsened	**4**	**4**	**5**
5	47	F	Autism spectrum disorder	Blonanserin patch	Galcanezumab	Marked response	Unchanged	**4**	**17**	**17**
6	51	F	Neurotic depression	Lormetazepam	Erenumab	Marked response	Unchanged	**4**	**6**	**12** c
7	64	F	PTSD	Escitalopram	Fremanezumab	Marked response	Unchanged	**4**	**13**	**13**

*Note*: “Marked response” indicates substantial patient‐reported reduction in headache frequency and/or severity sufficient to improve functional status, assessed through clinical interview. Psychiatric trajectory assessed over follow‐up period based on clinical documentation by board‐certified psychiatrists.

Abbreviations: F, female; M, male; PTSD, post‐traumatic stress disorder.

^a^ Temporary discontinuation due to rash; switched to alternative CGRP monoclonal antibodies with subsequent benefit.

^b^ Recurrence of headache attacks; switched to alternative CGRP monoclonal antibodies with subsequent benefit.

^c^ CGRP therapy discontinued after sustained improvement; patient maintained on conventional preventive medication with continued benefit.

## DISCUSSION

In this psychiatric outpatient cohort at a regional general hospital, 14.3% of patients carried a headache‐related insurance diagnosis, and nearly half of documented headache care occurred within psychiatry. Primary headache disorders were common, yet specialty referral was modest. These findings suggest that psychiatrists frequently serve as first‐line providers for headache management and that systematic headache assessment within psychiatry is warranted.

The predominance of nonspecific “headache” diagnoses in our cohort (56.4%) warrants particular attention. While this represents a methodological limitation, it simultaneously highlights a critical gap in psychiatric practice: systematic headache assessment and precise diagnostic classification are often not prioritized when psychiatric symptoms dominate clinical attention. This finding underscores our central clinical implication—that structured headache assessment tools and diagnostic approaches aligned with ICHD‐3 criteria are urgently needed in psychiatric settings. The discrepancy between routine clinical documentation and research‐grade headache classification reflects both a limitation of retrospective chart review and a call to action for improving headache care within psychiatry. Our exploratory CGRP case series indicates that psychiatric comorbidity does not necessarily preclude a favorable headache response to CGRP‐targeted therapy. All seven cases improved with CGRP monoclonal antibodies, though psychiatric symptoms did not uniformly improve. This pattern supports the clinical impression that the time course and mechanisms underlying headache relief versus mood/anxiety change may differ. Consequently, parallel mental‐health management (e.g., optimization of psychotropic regimens, psychotherapies, and sleep and lifestyle interventions) should accompany headache‐specific treatment.

These results align with prior literature showing high comorbidity between primary headaches and psychiatric disorders, and reinforce the risk that headache may be under‐recognized when overshadowed by prominent psychiatric presentations.^1^, ^2^, ^3^, ^4^ In practice, under‐recognition may lead to reliance on nonspecific analgesics, potential overuse, and delayed initiation of migraine‐specific therapies, thereby risking MOH and disability. Establishing clear care pathways—screening with tools such as HIT‐6/MIDAS when feasible, early differentiation of primary headache types, and timely initiation of evidence‐based acute and preventive treatments—may improve outcomes for this population.

Several studies have examined CGRP‐targeted therapies in patients with migraine who have comorbid psychiatric symptoms or diagnoses, but the evidence base remains limited and heterogeneous.^5^, ^6^, ^7^, ^8^, ^9^ Current evidence indicates that psychiatric comorbidity does not preclude a favorable headache response to CGRP‐targeted therapy. However, improvements in mood or anxiety symptoms are variable and may depend on baseline severity, the specific psychometric instruments used, and follow‐up duration. Our single‐center psychiatric outpatient cohort aligns with this pattern: robust headache improvement with CGRP agents was observed despite only modest or inconsistent change in psychiatric measures. These findings underscore the value of integrating headache management with concurrent, targeted mental‐health interventions rather than assuming that headache improvement will automatically translate into psychiatric remission. These results align with evidence that pain serves as a mediating factor between chronic conditions and psychiatric disorders.^10^ Implementing structured diagnostic‐therapeutic care pathways with systematic headache screening in psychiatric settings may help address this complex interplay.^11^, ^12^

### Future directions

To address the methodological limitations identified in this retrospective analysis, we have designed and obtained IRB approval for a prospective ambispective observational study with systematic collection of validated psychiatric assessment tools (PHQ‐9, GAD‐7, and HADS) and headache outcomes (HIT‐6, MIDAS^10^, and monthly migraine days via headache diaries). This ongoing study will enable rigorous quantitative analysis of the relationship between headache improvement and psychiatric symptom trajectories in patients receiving CGRP‐targeted therapy versus conventional preventive treatments, with systematic evaluation of psychotropic medication effects and lifestyle factors. Such prospective research is essential to establish evidence‐based guidelines for managing headache in psychiatric populations.

### Clinical implications

1. **Build headache‐aware psychiatric clinics:** integrate brief headache screening (HIT‐6/MIDAS), structured history‐taking, and red‐flag checks.
2. **Promote bidirectional referral** with neurology/headache specialists while retaining capacity for first‐line management within psychiatry.
3. **Use CGRP‐targeted therapy when indicated** for migraine after appropriate evaluation—even under psychiatric comorbidity—and pair with mental‐health interventions.
4. **Mitigate medication overuse** by auditing acute‐medication days and providing education on appropriate use.

### Strengths and limitations

Strengths include a clearly defined hospital‐wide psychiatric cohort and a combined description of overall prescribing patterns with an exploratory CGRP case series. This study has several limitations. First, the diagnostic and assessment methods were not fully standardized. Headache diagnoses were based on insurance billing codes used in routine clinical practice, which often do not correspond precisely to ICHD‐3 classifications. Psychiatric outcomes were evaluated through narrative clinical documentation rather than validated symptom scales such as patient health questionnaire‐9 (PHQ‐9) or GAD‐7, and standardized headache assessments (e.g., HIT‐6, MIDAS) were not systematically administered.

Second, this was a single‐center retrospective study, and the quality of documentation varied across cases. Psychotropic medication regimens and lifestyle factors (e.g., sleep quality, caffeine intake, and stress levels), which may influence both headache and psychiatric symptoms, were heterogeneous and not systematically analyzed. These factors limit the ability to control for confounders.

Third, the exploratory CGRP case series involved a small number of patients and limited observation periods, which restricts the generalizability of the findings. Prospective studies with larger sample sizes, longer follow‐up, and standardized headache and psychiatric assessments are warranted.

## CONCLUSIONS

Among psychiatric outpatients at a regional general hospital, primary headaches were common and often managed within psychiatry. CGRP monoclonal antibodies provided headache relief under psychiatric comorbidity, while psychiatric symptoms showed modest or inconsistent medium‐term change. Early, structured headache assessment and cross‐specialty pathways—alongside parallel mental‐health care—are essential to optimize outcomes for this vulnerable population. Future prospective research employing validated headache assessment tools (HIT‐6, MIDAS, and headache diaries) and standardized psychiatric scales should be prioritized to rigorously characterize the bidirectional relationship between headache and psychiatric outcomes in this vulnerable population and to establish evidence‐based treatment algorithms.

## AUTHOR CONTRIBUTIONS

Kyohei Otani conceived and designed the study, conducted data analysis, and drafted the manuscript.　Nobuyasu Imbe and Ryota Shindo contributed to data collection and critical revision of the manuscript. All authors read and approved the final version of the manuscript.

## CONFLICT OF INTEREST STATEMENT

The authors declare no conflicts of interest.

## ETHICS APPROVAL STATEMENT

This study was approved by the Institutional Review Board of Kakogawa Central City Hospital (No. 2024‐53).

## PATIENT CONSENT STATEMENT

Consent was waived due to the retrospective design with de‐identified data.

## CLINICAL TRIAL REGISTRATION

N/A.

## Data Availability

De‐identified data are available from the corresponding author on reasonable request and with institutional permission.

## References

[1] Minen MT , Begasse De Dhaem O , Kroon Van Diest A , Powers S , Schwedt TJ , Lipton R , et al. Migraine and its psychiatric comorbidities. J Neurol Neurosurg Psychiatry. 2016;87:741–749. 26733600 10.1136/jnnp-2015-312233

[2] Caponnetto V , Deodato M , Robotti M , Koutsokera M , Pozzilli V , Galati C , et al. Comorbidities of primary headache disorders: a literature review with meta‐analysis. J Headache Pain. 2021;22:71. 34261435 10.1186/s10194-021-01281-z PMC8278743

[3] Song TJ , Cho SJ , Kim WJ , Yang KI , Yun CH , Chu MK . Anxiety and depression in tension‐type headache: a population‐based study. PLoS One. 2016;11:e0165316. 27783660 10.1371/journal.pone.0165316 PMC5082613

[4] Hommer R , Lateef T , He JP , Merikangas K . Headache and mental disorders in a nationally representative sample of American youth. Eur Child Adolesc Psychiatry. 2022;31:39–49. 33721086 10.1007/s00787-020-01599-0 PMC8691207

[5] de Vries Lentsch S , van der Arend BWH , de Boer I , van Zwet EW , MaassenVanDenBrink A , Terwindt GM . Depression and treatment with anti‐calcitonin gene related peptide (CGRP) (ligand or receptor) antibodies for migraine. Eur J Neurol. 2024;31(2):e16106. 37847221 10.1111/ene.16106 PMC11235758

[6] Della Vecchia A , De Luca C , Becattini L , Curto L , Ferrari E , Siciliano G , et al. Beyond pain relief: unveiling the multifaceted impact of anti‐CGRP/R mAbs on comorbid symptoms in resistant migraine patients. Biomedicines. 2024;12(3):677. 38540290 10.3390/biomedicines12030677 PMC10968025

[7] Bottiroli S , De Icco R , Vaghi G , Pazzi S , Guaschino E , Allena M , et al. Psychological predictors of negative treatment outcome with erenumab in chronic migraine: data from an open label long‐term prospective study. J Headache Pain. 2021;22(1):114. 34600468 10.1186/s10194-021-01333-4 PMC8487575

[8] Torres‐Ferrús M , Gallardo VJ , Alpuente A , Caronna E , Giné‐Ciprés E , Pozo‐Rosich P . Improvement of migraine depressive symptoms is not related to headache frequency: exploring the impact of anti‐CGRP therapies. Cephalalgia. 2024;44(2):3331024231222923. 38307497 10.1177/03331024231222923

[9] Lipton RB , Ramirez Campos V , Roth‐Ben Arie Z , Galic M , Mitsikostas D , Tassorelli C , et al. Fremanezumab for the treatment of patients with migraine and comorbid major depressive disorder: the UNITE randomized clinical trial. JAMA Neurol. 2025;82(6):560–569. 40323613 10.1001/jamaneurol.2025.0806 PMC12053796

[10] Sauro KM , Rose MS , Becker WJ , Christie SN , Giammarco R , Mackie GF , et al. HIT‐6 and MIDAS as measures of headache disability in a headache referral population. Headache. 2010;50:383–395. 19817883 10.1111/j.1526-4610.2009.01544.x

[11] Ma Y , Xiang Q , Yan C , Liao H , Wang J . Relationship between chronic diseases and depression: the mediating effect of pain. BMC Psychiatry. 2021;21:436. 34488696 10.1186/s12888-021-03428-3 PMC8419946

[12] Cevoli S , Barbanti P , Finocchi C , Benedan L , Mariani P , Orthmann N , et al. Improvement in diagnostic–therapeutic care pathways for women with migraine: an Italian Delphi panel. Front Neurol. 2024;15:1436258. 39301474 10.3389/fneur.2024.1436258 PMC11412109

